# Enhanced 2,3-Butanediol Production by Optimizing Fermentation Conditions and Engineering *Klebsiella oxytoca* M1 through Overexpression of Acetoin Reductase

**DOI:** 10.1371/journal.pone.0138109

**Published:** 2015-09-14

**Authors:** Sukhyeong Cho, Taeyeon Kim, Han Min Woo, Jinwon Lee, Yunje Kim, Youngsoon Um

**Affiliations:** 1 Clean Energy Research Center, Korea Institute of Science and Technology (KIST), Seoul, Republic of Korea; 2 Interdisciplinary Program in Agriculture Biotechnology, College of Agriculture and Life science, Seoul National University, Seoul, Republic of Korea; 3 Clean Energy and Chemical Engineering, Korea University of Science and Technology, Daejeon, Republic of Korea; 4 Department of Chemical and Biomolecular Engineering, Sogang University, Seoul, Republic of Korea; University of Huddersfield, UNITED KINGDOM

## Abstract

Microbial production of 2,3-butanediol (2,3-BDO) has been attracting increasing interest because of its high value and various industrial applications. In this study, high production of 2,3-BDO using a previously isolated bacterium *Klebsiella oxytoca* M1 was carried out by optimizing fermentation conditions and overexpressing acetoin reductase (AR). Supplying complex nitrogen sources and using NaOH as a neutralizing agent were found to enhance specific production and yield of 2,3-BDO. In fed-batch fermentations, 2,3-BDO production increased with the agitation speed (109.6 g/L at 300 rpm vs. 118.5 g/L at 400 rpm) along with significantly reduced formation of by-product, but the yield at 400 rpm was lower than that at 300 rpm (0.40 g/g vs. 0.34 g/g) due to acetoin accumulation at 400 rpm. Because AR catalyzing both acetoin reduction and 2,3-BDO oxidation in *K*. *oxytoca* M1 revealed more than 8-fold higher reduction activity than oxidation activity, the engineered *K*. *oxytoca* M1 overexpressing the *budC* encoding AR was used in fed-batch fermentation. Finally, acetoin accumulation was significantly reduced by 43% and enhancement of 2,3-BDO concentration (142.5 g/L), yield (0.42 g/g) and productivity (1.47 g/L/h) was achieved compared to performance with the parent strain. This is by far the highest titer of 2,3-BDO achieved by *K*. *oxytoca* strains. This notable result could be obtained by finding favorable fermentation conditions for 2,3-BDO production as well as by utilizing the distinct characteristic of AR in *K*. *oxytoca* M1 revealing the nature of reductase.

## Introduction

2,3-butanediol (2,3-BDO) is an important chemical intermediate in numerous industrial applications [1, 2]. Methyl ethyl ketone (an industrial solvent) and 1,3-butadiene (a building block for synthetic rubber) can be converted by the dehydration of 2,3-BDO [3, 4]. Furthermore, 2,3-BDO is used in the manufacture of printing inks, synthetic perfumes, softening and moistening agents; as a solvent for resins and lacquers; as a carrier for drugs and pharmaceuticals [5, 6]; and as an anti-freeze agent due to its low freezing point. Although chemical synthetic methods are available for the production of 2,3-BDO and its derivatives, there have been considerable efforts and progress in the development of biomass-based 2,3-BDO production because of the depletion of petroleum oil and increasing concern for global warming.

A number of microorganisms are able to produce 2,3-BDO via the mixed acid-butanediol pathway from various substrates. Until now, *Klebsiella pneumoniae*, *K*. *oxytoca*, *Serratia marcescens*, *Enterobacter aerogenes*, *and Paenibacillus polymyxa* have been classified as key 2,3-BDO producers [7–11]. Among them, *Klebsiella* species have shown the greatest potential for industrial 2,3-BDO production due to the capability for using diverse substrates and its cultural adaptability [12].

Especially, *K*. *oxytoca* has an advantage on downstream processing over *K*. *pneumoniae* owing to much less formation of capsular polysaccharide [13]. Several strategies such as altering agitation speeds and reducing by-product formation by metabolic engineering have been developed to enhance 2,3-BDO production with *K*. *oxytoca*. The effects of aeration on cell growth, 2,3-BDO production and by-product formation have been studied [14]. Ji et al. proposed a two-stage agitation speed control strategy in batch fermentation to obtain high concentration, high yield, and high productivity using a random mutant *K*. *oxytoca* ME-UD-3 [3]. In fed-batch fermentation, instead of applying a two-stage agitation speed control strategy, Ji et al. inactivated the *aldA* gene (encoding aldehyde dehydrogenase) [3] in order to block ethanol pathway, which competes with the 2,3-BDO synthesis for pyruvate and nicotinamide adenine dinucleotide coenzyme (NADH), resulting in the improvement of 2,3-BDO production (130 g/L) with the productivity of 1.63 g/L/h and yield of 0.48 g/g glucose [8]. Jantama et al. reported the highest yield of 2,3-BDO (0.49 g/g) by metabolic engineered *K*. *oxytoca* KMS005 Δ*adhE*Δ*ack-pta*Δ*ldhA* to minimize by-products formation (117.4 g/L, 1.2 g/L/h) [15]. Combining a two-stage agitation speed control strategy and metabolic engineering to minimize lactic acid and formic acid production was attempted using *K*. *oxytoca* KCTC12133BP Δ*ldhA* Δ*pflB* in fed-batch fermentation and 113 g/L of 2,3-BDO was obtained [16]. Although those previous studies have contributed to high 2,3-BDO production by *K*. *oxytoca*, further investigation on fermentation conditions and metabolic engineering strategy is required in order to understand the factors affecting 2,3-BDO production and consequently to facilitate the industrial use of *K*. *oxytoca* to obtain higher 2,3-BDO production.

We previously reported a newly isolated bacterium, designated as *K*. *oxytoca* M1, which was capable of selectively producing 2,3-BDO and acetoin as a major product at 30°C and 37°C, respectively [17]. In this study, high production of 2,3-BDO from glucose was investigated using *K*. *oxytoca* M1 based on the fact that it produced 2,3-BDO with high yields and low levels of by-product formation in the batch fermentation compared to other *K*. *oxytoca* strains. Effects of complex nitrogen source, neutralizing agent, and agitation speed on 2,3-BDO production were investigated to find fermentation conditions favoring 2,3-BDO production. Further enhancement of 2,3-BDO production during fed-batch fermentation was attempted by overexpressing the *budC* gene encoding acetoin reductase (AR) in *K*. *oxytoca* M1, which revealed the nature of reductase with much higher acetoin reduction activity than 2,3-BDO oxidation activity. Subsequently, 2,3-BDO production was remarkably enhanced up to 142 g/L, which is the highest 2,3-BDO concentration obtained by *K*. *oxytoca* strains to date.

## Materials & Methods

### Microorganisms and media

The strain for 2,3-BDO fermentation was *K*. *oxytoca* M1 deposited in the Korean Culture Center of Microorganisms (KCCM) as KCCM 11177P. The *budC* from *K*. *oxytoca* M1 was cloned into pUC18CM in order to construct pUC18CM-*budC* and recombinant strain *K*. *oxytoca* M1 harboring pUC18CM-*budC* was generated by electroporation as described in the previous study [17].

Before batch fermentation, a pre-culture was grown in Luria–Bertani (LB) medium. The defined medium used for fermentation contained (per L of distilled water): K_2_HPO_4_ 13.7 g, KH_2_PO_4_ 2 g, (NH_4_)_2_HPO_4_ 3.3 g, (NH_4_)_2_SO_4_ 6.6 g, MgSO_4_ 7H_2_O 0.25 g, FeSO_4_ 7H_2_O 0.05 g, ZnSO_4_ 7H_2_O 0.001 g, MnSO_4_ H_2_O 0.01 g, CaCl_2_ 2H_2_O 0.01 g, and EDTA 0.05 g. Glucose was added to the defined medium as needed. The modified medium used for 2,3-BDO production consisted of the defined medium with 5 g/L yeast extract and 10 g/L casamino acid.

### Flask experiments with various glucose concentrations and complex nitrogen sources

Seed culture was grown overnight in LB medium at 30°C and 200 rpm. Then the seed culture was inoculated into a 100 mL Erlenmeyer flask (5% v/v) containing 20 mL of medium for flask experiments with various glucose concentrations and complex nitrogen sources. Glucose was added in the defined medium as needed (40~100 g/L) to investigate metabolite production patterns (initial pH 6.5, 30°C, 200 rpm, 48 hours). To investigate the effect of complex nitrogen sources, the defined medium and the modified medium containing complex nitrogen sources were used with 60 g/L of glucose. The complex nitrogen sources used were 5 g/L yeast extract (Difco) and 10 g/L casamino acid (Difco). Samples were taken after 12 hours cultivation at 200 rpm and 30°C to measure dry cell weight and 2,3-BDO concentration. All experiments were performed in triplicate.

### Acetoin reductase activity assay

*K*. *oxytoca* M1 and *K*. *oxytoca* M1 (pUC18CM-*budC*) were cultivated in LB medium for 12 hours and they were inoculated into 20 mL of the defined medium with 40 g/L of glucose and shaken at 200 rpm for 24 hours at 30°C. Harvested cell pellet was suspended in 1 mL lysis buffer containing 50 mM NaH_2_PO_4_, 300 mM NaCl, 10 mM imidazole, and 1 mg/mL of lysozyme (Sigma, Steinheim, Germany) at pH 8.0 and the mixture was incubated on ice for 1 hour. After centrifugation of cell lysate at 13000 rpm at 4°C, the supernatant was collected. Before measurement of the AR activity, buffer was exchanged to 100 mM of phosphate buffer (pH 7.0) using an Amicon ultrafiltration unit with a 10 kDa cut-off (Millipore, Billerica, MA, USA) and the crude protein, which was larger than 10 kDa including the acetoin reductase (27 kDa), was used for the AR activity measurement. The concentration of total crude protein was measured using a spectrophotometer (Cary 60 UV-Vis, Agilent Technologies, USA) using Bradford method [18].

Acetoin reduction activity of AR was determined spectrophotometrically by measuring the consumed NADH at 340 nm using the molar extinction coefficient of NADH over 5 minutes (Cary 60 UV-Vis, Agilent Technologies, USA) [19]. The reaction mixtures containing 50 mM sodium phosphate buffers (pH 6) with 0.15 mM NADH and 1.0 mM acetoin were incubated for 5 minutes at 30°C. After adding crude protein (100 μg/mL), the reaction was started. One unit of AR activity was defined as the amount of crude protein required to reduce 1 μmol of NADH in 1 min. All experiments were performed in triplicate.

To determine 2,3-BDO oxidation activity of AR, crude proteins from *K*. *oxytoca*M1 parent strain and *K*. *oxytoca* M1 harboring pUC18CM-*budC* were extracted as described above. Enzyme assay was done at 30°C and pH 6.0 with 0.15 mM NAD^+^ and 1.0 mM 2,3-BDO. 2,3-BDO oxidation activity of AR was analyzed by measuring the generated NADH in absorbance at 340 nm. All experiments were performed in triplicate.

### Fed-batch fermentation

Fed-batch fermentations were conducted in a 3 L- stirred fermenter (Fermentec FMT ST, Korea) with an initial working volume of 1 L. For seed culture preparation, *K*. *oxytoca* M1 and *K*. *oxytoca* M1 (pUC18CM-*budC*) were inoculated into 100 mL fresh defined medium with 60 g/L of glucose and cultivated on rotary shaker with 200 rpm at 30°C for 10 hours. *K*.*oxytoca* M1 harboring pUC18CM-*budC* plasmid was cultured with 25 μg/ mL of chloramphenicol to maintain the plasmid. Seed culture was then inoculated into the fermentation medium (10% v/v). All cultivations were carried out at 30°C and pH was controlled at 6 by automatically adding 5 N KOH or 5 N NaOH. The aeration rate was 1.0 volume of air per volume of liquid per minute (vvm), while the agitation speed was controlled at 200, 300 and 400 rpm. Fed-batch fermentation was performed with the initial glucose concentration of 80~100 g/L and the feeding solution containing 600 g/L glucose and the composition of the modified medium was added to supply glucose when needed. Samples were collected periodically to determine the cell mass, concentrations of glucose, 2,3-BDO and by-products.

### Analytical methods

Dry cell weight (DCW, g/L) was calculated from optical density at 600 nm (OD_600_) using calibration curve of OD_600_ vs. dry cell weight of *K*. *oxytoca* M1. The OD_600_ of the broth was measured using UV- visible spectrophotometer (Cary 60 UV-Vis, Agilent Technologies, USA) with appropriate dilution.

The concentration of 2,3-BDO, acetoin, acetic acid, and ethanol were measured using a gas chromatography (Shimadzu GC-2010, Kyoto, Japan) equipped with a flame ionized detector (FID) and an HP-INNOWax column (30 m X 0.32 mm X 0.25 μm) under the conditions described previously [17, 20]. Glucose, lactic acid, and succinic acid were analyzed using a high-performance liquid chromatography (HPLC) (Agilent 1260, Waldbronn, Germany) equipped with a refractive index detector (RID) and an Aminex HPX-87 H Ion Exclusion Column (300 mm X7.8 mm, Bio-Rad, Hercules, CA, USA) under the following conditions: sample volume of 20 μl, mobile phase of 5 mM H_2_SO_4_, flow rate of 0.5 mL/min, and column temperature of 50°C. All samples tested were preliminary purified through a membrane filter (0.45 μm pore size, Millipore, USA).

## Results and Discussion

### Metabolite production of *K*. *oxytoca* M1 in batch cultures

In the previous study, *K*. *oxytoca* M1 was assessed for its ability to utilize various sugars [17]. Because the highest yield of 2,3-BDO was achieved using glucose, further study was performed with glucose as a carbon source for 2,3-BDO production. Metabolite production of *K*. *oxytoca* M1 was investigated using flask cultures with 40~100 g/L glucose concentrations. When 42 g/L of glucose was used, the concentration of 2,3-BDO increased until 24 hours (up to 11.7 g/L) and then slightly decreased after glucose was completely consumed (S1 Fig). On the other hand, acetoin concentration increased after 24 hours, implying the reverse conversion of 2,3-BDO to acetoin in the absence of carbon source (i.e., glucose) as reported in the previous studies [21, 22]. The metabolite profile from 90 g/L of glucose is shown in S1 Fig as a representative result presenting no reverse conversion of 2,3-BDO to acetoin. As shown in Table 1, 2,3-BDO concentration, yield, and productivity were similar with initial glucose concentrations ranging from 69.3 to 104.5 g/L and they were significantly higher than those with 42.3 g/L glucose. Yields were in a range of 0.35~0.38 g/g, which were much higher than the previously reported 2,3-BDO yields from batch cultures using the wild type of *K*. *oxytoca* ATCC43863 (0.15~0.24 g/g) [13] and *K*. *oxytoca* KCTC12133BP (0.18 g/g) [16]. *K*. *oxytoca* ME-303, a random mutated strain producing a low level of acids, showed a 2,3-BDO yield of 0.39 g/g along with the production of 2.9 g/L of mixed acid, 5.5 g/L of acetoin, and 3.3 g/L of ethanol after consuming 100 g/L glucose [23]. In the case of *K*. *oxytoca* M1, total acid production was comparable to that of *K*. *oxytoca* ME-303, while the amounts of acetoin and ethanol produced were even lower than those of *K*. *oxytoca* ME-303. Based on high 2,3-BDO yield values and low by-product formation, *K*. *oxytoca* M1 was further examined as a potential strain for high 2,3-BDO production.

**Table 1:** Metabolite production by *K*. *oxytoca* M1 using various glucose concentrations in flask cultures (initial pH 6.5, 30°C, 200 rpm, 48 hours cultivation).

Initial glucose (g/L)	42.3	69.3	90.0	104.5
Consumed glucose (g/L)	42.3±0.99	69.3±3.89	75.6±6.79	78.1±3.89
2,3-BDO (g/L)	10.38±0.28	26.17±0.07	26.75±4.21	27.67±1.52
Acetoin (g/L)	3.82±1.02	1.39±0.67	1.20±0.71	1.13±0.80
Acetic acid (g/L)	1.11±0.16	0.44±0.08	0.38±0.01	0.38±0.08
Lactic acid (g/L)	0.06±0.01	0.19±0.08	0.50±0.11	0.87±0.07
Succinic acid (g/L)	0.09±0.04	0.67±0.15	0.10±0.32	1.10±0.17
Ethanol (g/L)	0.66±0.01	1.62±0.02	1.67±0.02	1.86±0.02
2,3-BDO yield (g/g)	0.25±0.01	0.38±0.02	0.36±0.09	0.35±0.04
2,3-BDO productivity (g/L/h)	0.22±0.01	0.55±0.01	0.56±0.09	0.58±0.04

### Effect of complex nitrogen sources on 2,3-BDO production

**Fig 1:**
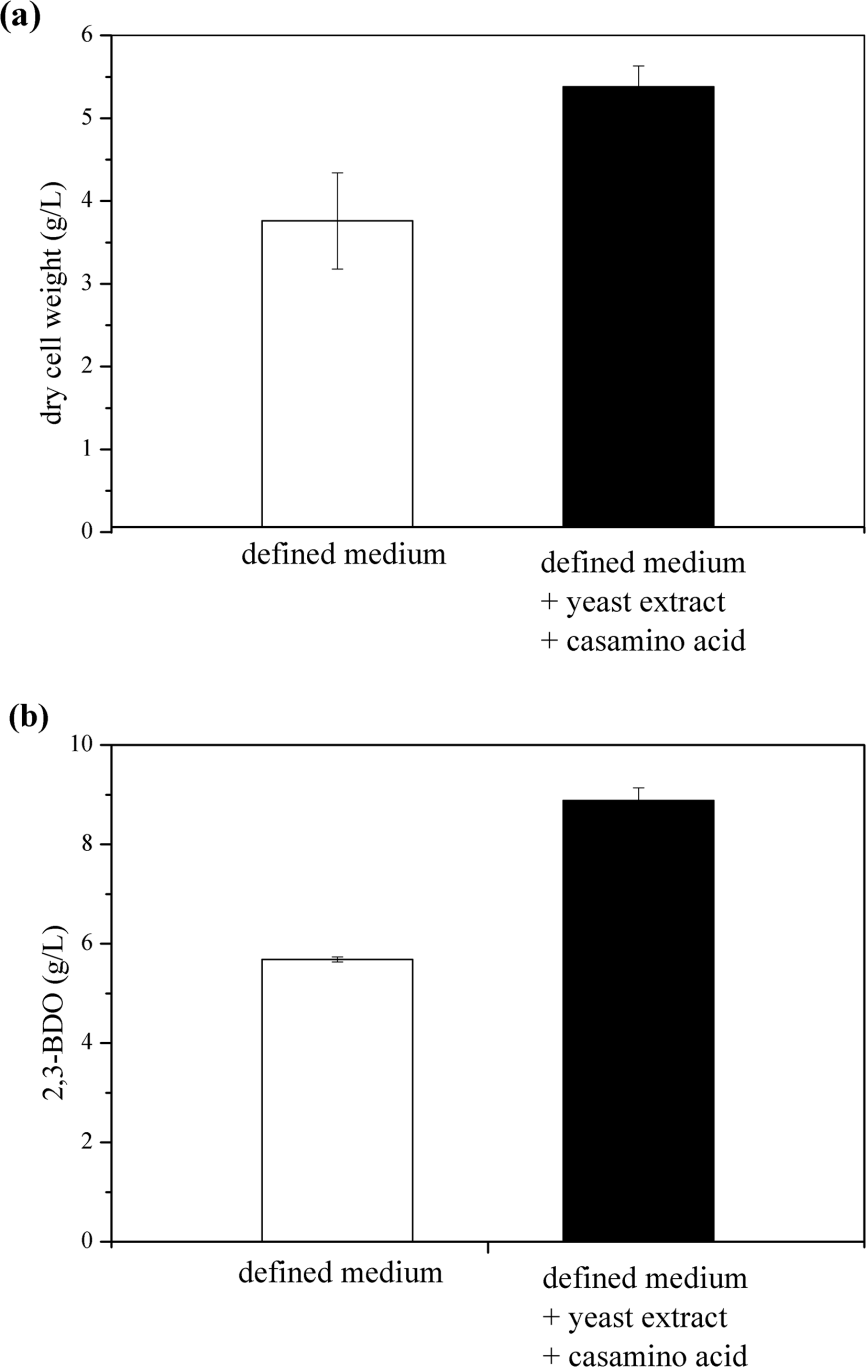
Effect of complex nitrogen source on 2,3-BDO fermentation. (a) dry cell weight and (b) 2,3-BDO production. *K*. *oxytoca* M1 was grown for 12 hours at 30°C and 200 rpm in an Erlenmeyer flask containing 60 g/L glucose. Growth medium used were the defined medium (□) and the defined medium supplemented with 5 g/L yeast extract and 10 g/L casamino acid (i.e. the modified medium) (■).

Complex nitrogen sources have been used to enhance cell growth and 2,3-BDO production [10]. To investigate the effect of complex nitrogen sources on the fermentation of *K*. *oxytoca* M1, cell biomass and 2,3-BDO production in the defined medium were compared to those in the modified medium containing yeast extract (5 g/L) and casamino acid (10 g/L) using flask cultures with glucose as a carbon source. As shown in Fig 1, cell biomass and 2,3-BDO production were enhanced by 1.4 fold (3.8 g/L vs. 5.4 g/L) and 1.6 fold (5.7 g/L vs. 8.9 g/L), respectively, when *K*. *oxytoca* M1 was cultivated in the modified medium. Consequently, the specific 2,3-BDO production (g of 2,3-BDO production/ g of dry cell weight) in the modified medium was higher than in the defined medium by 1.1-fold, indicating that the modified medium slightly favored 2,3-BDO production in *K*. *oxytoca* M1. Considering that the amount of nitrogen source in the defined medium (nitrogen 2.1 g/L, supplemented as ammonium) is sufficient to form 20 g/L of cell biomass (assuming that a composition of cell biomass is C_5_H_7_O_2_N [24]), a lower cell biomass formation from the defined medium was not likely caused by N source limitation. *Klebsiella* sp. has a gene cluster for synthesis of 2,3-BDO known as the *budABC* operon. The *budA*, *budB*, and *budC* genes encode for acetolactate decarboxylase, acetolactate synthase, and acetoin reductase, respectively [25]. Acetolactate is a central common metabolite involved in not only 2,3-BDO synthesis pathway but also branched-chain amino acid (BCAA) biosynthesis pathway such as leucine, isoleucine, and valine [26, 27]. Acetolactate decarboxylase, which is responsible for converting α-acetolactate to acetoin, regulates BCAA biosynthesis by diverting the flux of α-acetolactate towards acetoin when BCAAs are present at a high concentration in *Lactococcus lactis* and *Streptococcus thermophiles* [26, 27]. These results suggest that the addition of complex nitrogen sources containing various amino acids such as yeast extract and casamino acid would stimulate the metabolic conversion of α-acetolactate to acetoin rather than BCAA synthesis, consequently improving specific 2,3-BDO production. Hence, the modified medium containing yeast extract and casamino acid was used for further study.

### Effect of neutralizing agents for pH control on 2,3-BDO production

To achieve high production of 2,3-BDO, fed-batch fermentation was carried out in this study. During the flask cultivation, 2,3-BOO production stopped as pH decreased to below 5 (data not shown); therefore, pH control was required in fed-batch fermentation to achieve high 2,3-BDO production. To choose a suitable neutralizing agent, KOH and NaOH which are the most conventional alkali solutions were used to control pH at 6 and 2,3-BDO productions were compared during the fed-batch fermentation. As shown in Table 2, there was no significant difference in cell growth regardless neutralizing agents, while pH-controlled fed-batch fermentation with the addition of 5N NaOH showed much higher glucose consumption (138.6 g/L vs. 128.4g/L) and 2,3-BDO production (50.6 g/L vs. 38.3 g/L) than the fed-batch fermentation to which KOH was added. In addition, the yield, productivity, and specific production of 2,3-BDO were higher in NaOH-added fed-batch fermentation than those with KOH-added fermentation. Na^+^ ion is known to be an important factor promoting the substrate permeation through the transmembrane Na^+^ gradient and K^+^ ion plays a role in pumping out Na^+^ ions as well as adjusting the intracellular acid-base balance [28]. It was previously reported that using a mixture of NaOH and KOH for controlling the pH level improved product formation than using of NaOH or KOH alone because the balanced amount of cations (Na^+^ and K^+^) could promote substrate uptake and pumping out Na^+^ ions [28]. In this study, *K*. *oxytoca* M1 was cultured in the medium containing 13.7 g/L K_2_HPO_4_ (78.6 mM) and 2 g/L KH_2_PO_4_ (14.7 mM), which was generally used in 2,3-BDO fermentation with *K*. *oxytoca* [3, 8]. By supplying 5 N NaOH to control pH at 6, Na^+^ and K^+^ in the fermentation broth might be balanced, resulting in higher 2,3-BDO production than that obtained with the addition of KOH for pH control. Therefore, 5 N NaOH was used as a suitable neutralizing agent to control the pH level for 2,3-BDO production by *K*. *oxytoca* M1.

**Table 2:** Comparison of products in fed-batch fermentation by *K*. *oxytoca* M1 using different neutralizing agents for pH control at 6.

Neutralizing agent	5 N KOH^1^	5 N NaOH^1^
Final dry cell weight (g/L)	7.7	8.0
Glucose consumption (g/L)	128.4	138.6
2,3-BDO (g/L)	38.3	50.6
Acetoin (g/L)	1.9	1.5
2,3-BDO yield (g/g)	0.30	0.37
2,3-BDO productivity (g/L/h)	1.06	1.40

^1^Fed-batch fermentation was carried out in a bioreactor containing 1 L medium containing glucose, yeast extract (5 g/L), and casamino acid (10 g/L), 200 rpm, 30°C for 36 hours.

### Effect of agitation speed on 2,3-BDO production by *K*. *oxytoca* M1

**Fig 2:**
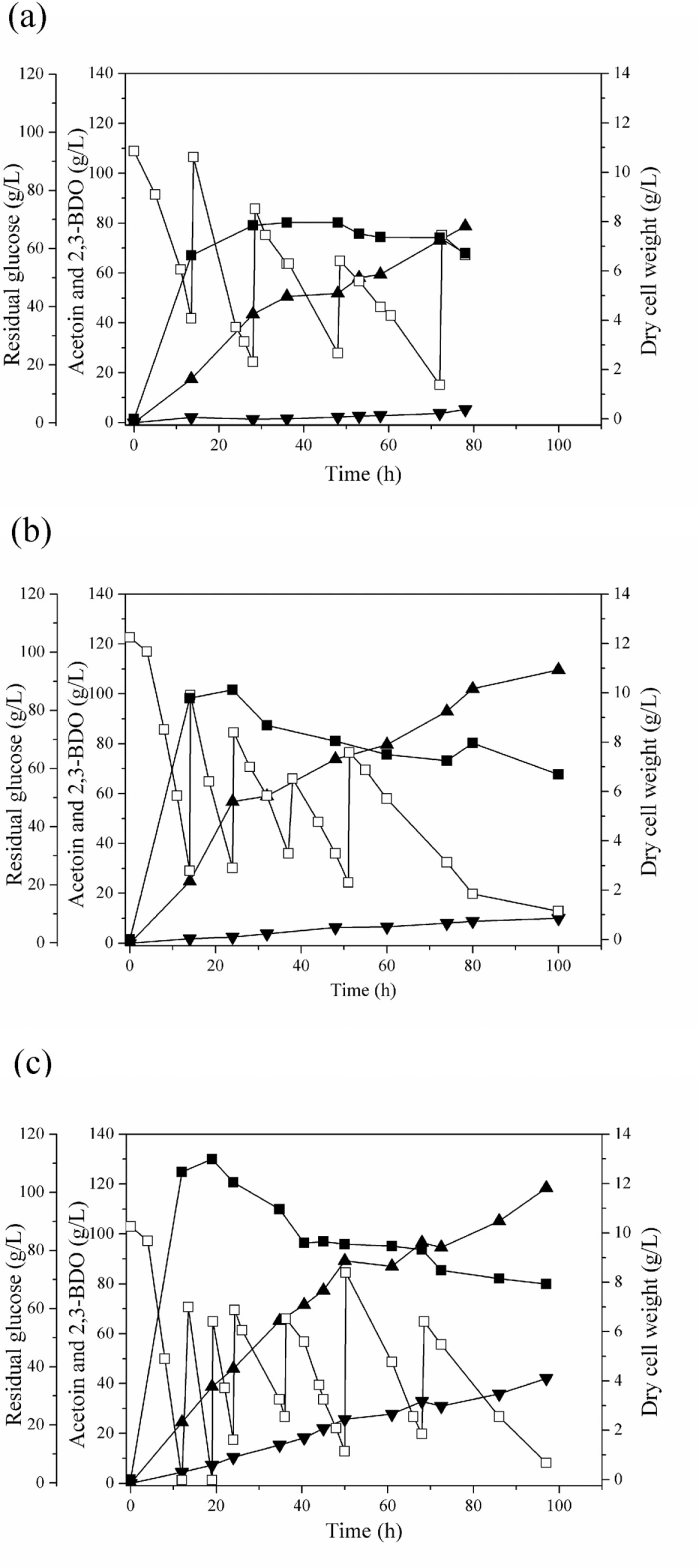
Time course of 2,3-butanediol production by *K*. *oxytoca* M1 at different agitation speeds. (a) 200 rpm, (b) 300 rpm, and (c) 400 rpm. Symbols indicate dry cell weight (■), residual glucose (□), 2,3-BDO (▲), and acetoin (▼).

Previous studies [3, 16] have demonstrated that the oxygen supply strategy is a critical factor for high production of 2,3-BDO. Because controlling DO level was not effective for 2,3-BDO production and cell growth [29], many studies on 2,3-BDO production have focused on controlling agitation speed as a simple and effective oxygen supply method [3, 16, 22, 29]. In particular, two-stage agitation speed depending on cellular status and cultivation stage was found to be effective for a high 2,3-BDO production [3, 16]. Hence, in this study, the optimum agitation speed was investigated for high production of 2,3-BDO using *K*. *oxytoca* M1. The time-course of 2,3-BDO fed-batch fermentation profiles of *K*. *oxytoca* M1 with various agitation speeds (200, 300, and 400 rpm) in a 3 L-bioreactor is shown in Fig 2. Dry cell weight increased rapidly during 24 hours of fermentation and the maximum dry cell weight values increased with agitation speeds (8.0 g/L at 200 rpm; 10.1 g/L at 300 rpm; and 13.0 g/L at 400 rpm). Moreover, a higher titer and productivity of 2,3-BDO were achieved after 80~85 hours of fermentation by increasing agitation speeds (78.8 g/L at 200 rpm; 102.1 g/L at 300 rpm; and 105.1 g/L at 400 rpm).

Because the oxygen supply at the agitation speed of 200 rpm seemed insufficient to support cell mass formation and 2,3-BDO production (Fig 2), the results obtained at 300 rpm and 400 rpm were further analyzed. As shown in Table 3, the amount of glucose consumption, dry cell weight, 2,3-BDO concentration, and 2,3-BDO productivity increased with the agitation speed, while 2,3-BDO yield deceased with the agitation speed. These results are similar to the previous studies: high oxygen supply tends to be favorable for cell mass formation and 2,3-BDO productivity, but 2,3-BDO yield decreases with agitation speed [3, 16]. Interestingly, the formation of ethanol and succinic acid were dramatically reduced by increasing the agitation speed from 300 to 400 rpm, and lactic acid was even not detected at 400 rpm (Table 3). This result might be caused by low DO levels at low agitation speeds, in which the formation of ethanol, succinic acid, and lactic acid generally increases [30, 31]. Considering that the production of ethanol, succinic acid, and lactic acid competes with 2,3-BDO biosynthesis pathway for carbon flux and NADH, a far lower concentration of those by-products at a higher agitation speed likely allowed the redistribution of carbon flux and NADH availability to 2,3-BDO synthesis pathway. Several studies have developed genetically engineered strains to reduce NADH-consuming by-product formation, consequently enhancing 2,3-BDO production. Ji et al. inactivated the *aldA* encoding acetaldehyde dehydrogenase to reduce ethanol production, resulting in enhancement of 2,3-BDO production [8]. Previous studies focusing on reducing lactic acid production have been also attempted to enhance 2,3-BDO production by deleting the *ldhA* encoding lactate dehydrogenase from *Enterobacter aerogenes* [10] and *K*. *oxytoca* KCTC12133BP [32]. In this study, NADH-consuming by-products were noticeably decreased by simply increasing agitation speed for the fed-batch fermentation of *K*. *oxytoca* M1, demonstrating an easy and convenient fermentation strategy in enlarging NADH availability for 2,3-BDO production.

**Table 3:** Comparison of fed-batch fermentation results of *K*. *oxytoca* M1 (pUC18CM-*budC*) with the parent strain at 300 rpm and 400 rpm.

Agitation speed	300 rpm		400 rpm	
Strain	*K*. *oxytoca* M1	*K*. *oxytoca* M1 (pUC18CM-*budC*)	*K*. *oxytoca* M1	*K*. *oxytoca* M1 (pUC18CM-*budC*)
Dry cell weight (g/L)	6.7	7.9	7.9	8.7
Glucose consumed (g/L)	271.7	273.4	346.1	343.3
2,3-BDO (g/L)	109.6	111.4	118.5	142.5
Acetoin(g/L)	10.0	6.5	42.1	18.0
Acetic acid (g/L)	4.3	2.7	7.3	11.0
Ethanol (g/L)	4.0	2.7	0.7	1.1
Succinic acid (g/L)	15.5	11.5	6.7	3.4
Lactic acid (g/L)	11.5	13.4	0.0	0.0
2,3-BDO productivity (g/L/h)	1.10	1.11	1.22	1.47
2,3-BDO yield (g/g)	0.40	0.41	0.34	0.42
Fermentation time (h)	100	100	97	97

Although the final 2,3-BDO concentration was enhanced and the formation of NADH-consuming by-product significantly decreased by increasing the agitation speed to 400 rpm, the 2,3-BDO yield was still much lower at 400 rpm than that of 300 rpm (0.34 g/g vs. 0.40 g/g). This result might be caused by a higher cell mass formation at the expense of 2,3-BDO production as well as acetoin accumulation at 400 rpm (Table 3). In particular, acetoin accumulation at 400 rpm was much higher than at 300 rpm (42.1 vs. 10.0 g/L). Even though previous fed-batch studies using *K*. *oxytoca* also showed the increase of acetoin accumulation up to 2.82~13.40 g/L when agitation speeds were increased [3, 16], a much higher acetoin accumulation phenomenon by *K*. *oxytoca* M1 is distinct from the previous *K*. *oxytoca* results. Considering that the amount of available NADH probably increased due to much lower production of NADH-consuming by-products (ethanol, succinic acid, and lactic acid), acetoin accumulation might result from a metabolic restraint converting acetoin to 2,3-BDO in *K*. *oxytoca* M1. AR encoded by the *budC* is a key enzyme in converting acetoin to 2,3-BDO through reduction and converting 2,3-BDO to acetoin through oxidation along with the conversion of coenzyme NADH to NAD^+^ and vice versa, respectively [33]. In general, to solve the acetoin accumulation problem in this study, overexpression of the *budC* can be applied in expectation of acetoin conversion to 2,3-BDO; however, according to previous studies, the *budC* overexpression was found not to be effective in improving 2,3-BDO production because AR also catalyzes 2,3-BDO oxidation reaction [22, 34]. Nevertheless, because the specific reactivity of AR to acetoin and 2,3-BDO would significantly influence acetoin and 2,3-BDO production profiles, acetoin reduction and 2,3-BDO oxidation activities of AR in *K*. *oxytoca* M1 were investigated to design a strategy for reducing acetoin accumulation and consequently enhancing 2,3-BDO yield.

### Investigation of AR activity for acetoin reduction and 2,3-BDO oxidation

**Fig 3:**
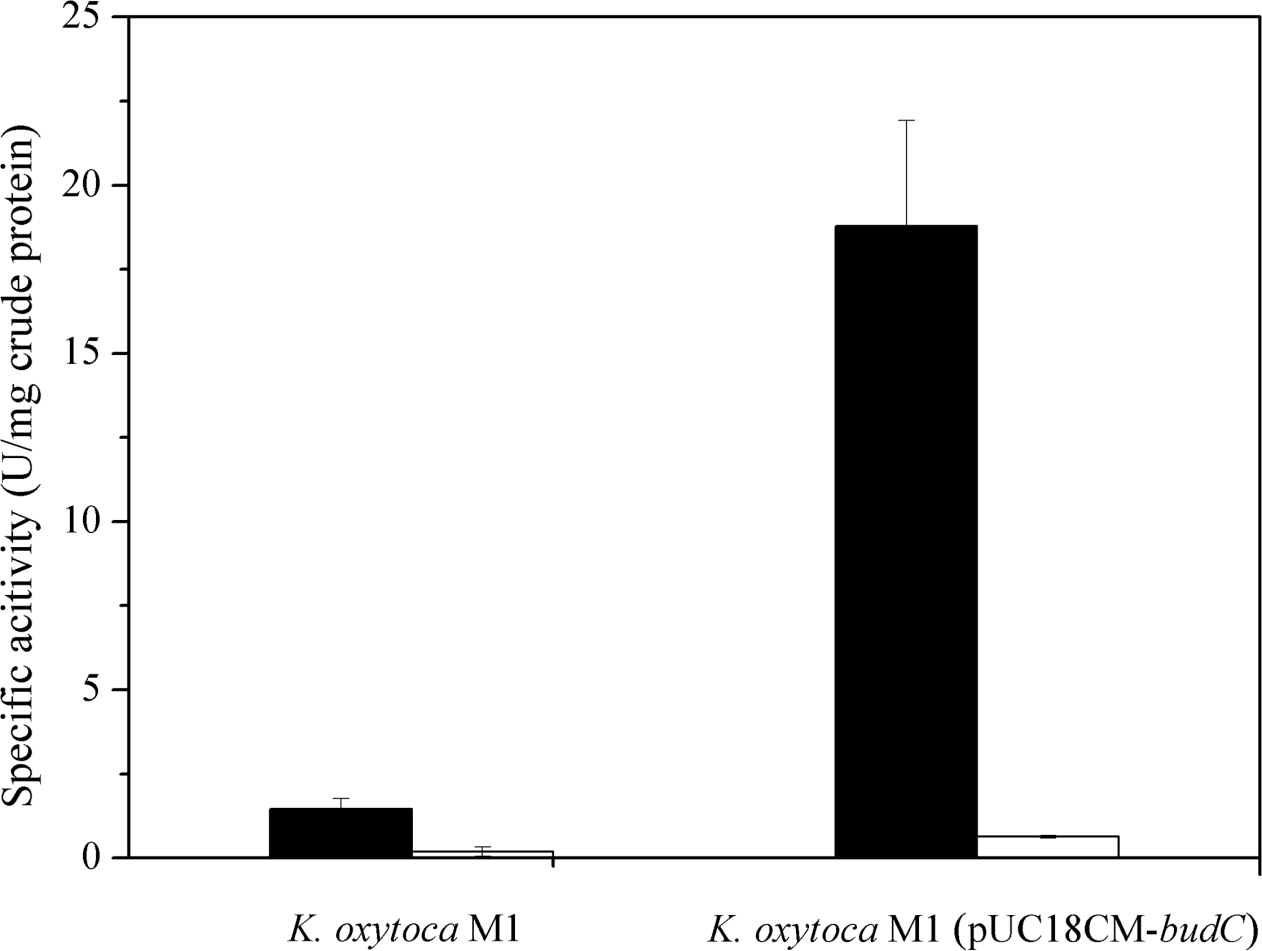
Acetoin reduction activity and 2,3-BDO oxidation activity of *K*. *oxytoca* M1 and *K*. *oxytoca* (pUC18CM-*budC*). Activity was analyzed with the crude protein extract of each strain. Bars indicate acetoin reduction activity (■) and 2,3-BDO oxidation activity (□). One unit of specific activity was defined as 1 μmol NADH consumed or generated per minute per 1 mg crude protein.

To estimate AR activity in *K*. *oxytoca* M1 toward acetoin reduction and 2,3-BDO oxidation, reductive reaction using acetoin and NADH as well as oxidative reaction using 2,3-BDO and NAD^+^ were evaluated using crude protein extract of *K*. *oxytoca* M1. As shown in Fig 3, acetoin reduction activity (1.46 U/mg) was much higher than 2,3-BDO oxidation activity (0.18 U/mg) in *K*. *oxytoca* M1, indicating that AR reactivity favored the reductive reaction (acetoin to 2,3-BDO). Further characterization of AR activity was attempted by cloning and purifying AR of *K*. *oxytoca* M1, but soluble expression of AR was not enough to purify AR for unknown reasons, despite of tens of expression trials under various conditions (data not shown). Instead, the crude protein extract from the recombinant *K*. *oxytoca* M1 harboring pUC18CM-*budC* was used to evaluate AR activity toward the reduction of acetoin and the oxidation of 2,3-BDO. As shown in Fig 3, the acetoin reduction activity of recombinant *K*. *oxytoca* M1 (pUC18CM-*budC*) was 12.9-fold higher than that of the parent strain (18.8 U/mg vs. 1.46 U/mg), indicating that AR of *K*. *oxytoca* M1 was functionally overexpressed in *K*. *oxytoca* M1 (pUC18CM-*budC*). When the reduction activity of AR was compared to the oxidation activity of AR in *K*. *oxytoca* M1 (pUC18CM-*budC*), the reduction activity was 29.8-fold higher than the oxidation activity (18.8 U/mg vs. 0.63 U/mg). Considering the much higher specific activity of AR toward acetoin reduction over 2,3-BDO oxidation in *K*. *oxytoca* M1 (8.1-fold) and *K*. *oxytoca* M1 (pUC18CM-*budC*) (29.8 fold), AR from *K*. *oxytoca* M1 would preferentially function as a reductase rather than an oxidase. In previous studies, several ARs (also named 2,3-BDO dehydrogenase) from various strains have been reported to exhibit 2–3 fold higher acetoin reduction activity in comparison to 2,3-BDO oxidation activity [33, 35, 36]. In contrast, even though AR activity was estimated with crude protein extract in this study, more than 8-fold higher acetoin reduction activity over 2,3-BDO oxidation activity in *K*. *oxytoca* M1 and *K*. *oxytoca* M1 (pUC18CM-*budC*)) is a remarkable characteristic. Hence, based on this result, the overexpression of the *budC* was attempted as a prospective approach to reduce acetoin accumulation and consequently increasing 2,3-BDO production during fed-batch fermentations.

### Improved 2,3-BDO production by the overexpression of the *budC*

**Fig 4:**
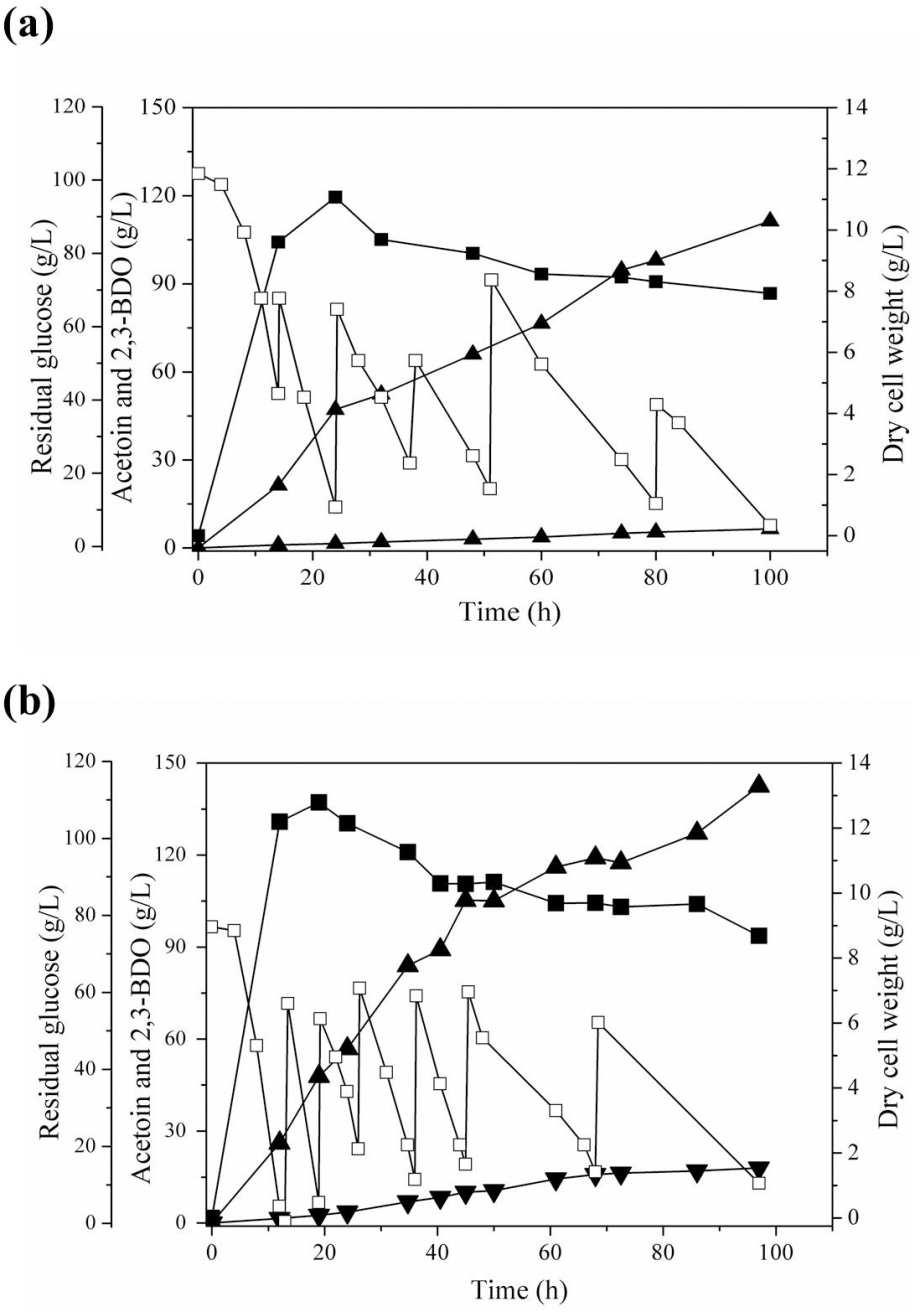
Time course of 2,3-BDO fermentation by *K*. *oxytoca* M1 (pUC18CM-*budC*) at different agitation speeds. (a) 300 rpm and (b) 400 rpm. Fed-batch fermentation was carried out with the medium containing 5 g/L yeast extract and 10 g/L casamino acid. Symbols indicate dry cell weight (■), residual glucose (□), 2,3-BDO (▲) and acetoin (▼).

To investigate whether the overexpression of the *budC* revealing the nature of reductase would enhance 2,3-BDO production and reduce acetoin accumulation, the time course of fed-batch fermentation profiles by *K*. *oxytoca* M1 harboring pUC18CM-*budC* was monitored at the agitation speeds of 300 rpm and 400 rpm (Fig 4). Table 3 compares the fed-batch fermentation results performed with the recombinant *K*. *oxytoca* M1 (pUC18CM-*budC*) and the parent strain at 300 rpm and 400 rpm.

In the case of fed-batch fermentation at 300 rpm, 2,3-BDO concentration, productivity, and yield with *K*. *oxytoca* M1 (pUC18CM-*budC*) were similar to those with the parent strain (Table 3). Although the overexpression of the *budC* was also effective to reduce acetoin accumulation at 300 rpm (10.0 g/L vs. 6.5 g/L), the reduction of acetoin accumulation did not seem to be enough to significantly influence 2,3-BDO production.

In contrast to 300 rpm, it was observed that by overexpression of the *budC* gene, the maximum 2,3-BDO titer reached 142.5 g/L with productivity of 1.47 g/L/h at the agitation speed of 400 rpm, which was 1.2-fold higher than that of *K*. *oxytoca* parent strain (118.5 g/L of 2,3-BDO with productivity of 1.22 g/L/h). Notably, acetoin accumulation with the recombinant strain was significantly reduced to 43% of that of the parent strain (42.1 g/L to 18.0 g/L). Considering the amount of 2,3-BDO production enhancement (24 g/L or 266 mM) and the reduced acetoin concentration (24.1 g/L or 272 mM) by *K*. *oxytoca* M1 (pUC18CM-*budC*) at 400 rpm, it implied that the overexpression of the *budC* at 400 rpm was effective in the conversion of acetoin to 2,3-BDO. Hence, owing to the reduced production of acetoin, the yield of 2,3-BDO using *K*. *oxytoca* M1 (pUC18CM-*budC*) (0.42 g/g) increased up to 1.2-fold higher than that of the parent strain at 400 rpm, overcoming the low yield problem with the parent strain at 400 rpm. The overexpression of the *budC* also caused an increase of cell mass formation, positively affecting the productivity of 2,3-BDO. Interestingly, although a two-stage agitation speed control strategy was reported to be effective to enhance 2,3-BDO production for *K*. *oxytoca* strains [3, 16], 2,3-BDO production by *K*. *oxytoca* M1 and *K*. *oxytoca* M1 (pUC18CM-*budC*) did not increase when the agitation speed was altered from 400 rpm to 300 rpm (96~114.7 g/L 2,3-BDO production after 131 hours).

Until now, Ji et al. [8] reported the highest production of 2,3-BDO (130 g/L) by *K*. *oxytoca* with the yield of 0.48 g/g glucose and the productivity of 1.63 g/L/h through insertional inactivation of the acetaldehyde dehydrogenase gene in the randomly mutated *K*. *oxytoca* ME-UD-3. Jantama et al. eliminated the alcohol dehydrogenase E (*adhE*), acetate kinase A-phosphotransacetylase (*ack-pta*), and lactate dehydrogenase A (*ldhA*) genes in order to reduce by-products, resulting in the improved 2,3-BDO production (117.4 g/L, 0.49 g/g glucose, 1.2 g/L/h) [15]. Park et al. [16] also achieved high concentration, yield, and productivity of 2,3-BDO (113 g/L, 0.45 g/g glucose, 2.1 g/L/h) using *K*. *oxytoca* KCTC12133BP Δ*ldhA* Δ*pflB* and a two-phase of agitation speed strategy. In this study, the carbon flux to 2,3-BDO during the fed-batch fermentation was significantly enhanced at the expense of NADH-consuming by-products at a high agitation speed (400 rpm) and through the overexpression of the *budC* gene (encoding AR) in *K*. *oxytoca* M1. Finally, a new record of high 2,3-BDO concentration was achieved (142.5 g/L) with the yield of 0.42 g/g and productivity of 1.47 g/L/h. This result could be successfully accomplished by recognizing and utilizing the distinct characteristic of AR activity in *K*. *oxytoca* M1 exhibiting a much higher acetoin reduction activity in comparison with 2,3-BDO oxidation.

## Conclusions

In this study, *K*. *oxytoca* M1 was investigated as a potential effective 2,3-BDO producer. By combining the following strategies: i) supplying complex nitrogen sources and using NaOH as a neutralizing agent; ii) the fed-batch fermentation at a high agitation speed (400 rpm); and iii) the overexpression of the *budC* gene (encoding AR) in *K*. *oxytoca* M1, 2,3-BDO production significantly increased up to 142.5 g/L with a yield of 0.42 g/g and productivity of 1.47 g/L/h, while formation of by-products such as ethanol, lactic acid, succinic acid, and acetoin decreased. In particular, unlike the results of previous studies, this study found that the overexpression of the *budC* was effective in the enhancement of 2,3-BDO production at the expense of acetoin because the AR from *K*. *oxytoca* M1 exhibited a distinct high reaction activity toward acetoin reduction in comparison with 2,3-BDO oxidation activity. To the best of our knowledge, the 2,3-BDO concentration achieved in this study is a new record in 2,3-BDO production with *K*. *oxytoca* strain. The results presented in this study demonstrate the feasibility of achieving high production of 2,3-BDO with *K*. *oxytoca* M1 suitable for industrial use.

## Supporting Information

S1 Fig Time course of 2,3-BDO and acetoin production by *K*. *oxytoca* M1 in 100 mL Erlenmeyer flask at 30°C using different initial glucose concentration. (a) 42.3 g/L of glucose, (b) 90.0 g/L of glucose. The symbols were used: residual glucose (■), 2,3-BDO (▲) and acetoin.
(TIF)
